# Immune Response to Third Dose BNT162b2 COVID-19 Vaccine Among Kidney Transplant Recipients—A Prospective Study

**DOI:** 10.3389/ti.2022.10204

**Published:** 2022-04-21

**Authors:** Dafna Yahav, Ruth Rahamimov, Tiki Mashraki, Naomi Ben-Dor, Tali Steinmetz, Timna Agur, Boris Zingerman, Michal Herman-Edelstein, Shelly Lichtenberg, Haim Ben-Zvi, Erez Bar-Haim, Hila Cohen, Shahar Rotem, Uri Elia, Ili Margalit, Benaya Rozen Zvi

**Affiliations:** ^1^ Infectious Diseases Unit, Rabin Medical Center, Petah-Tikva, Israel; ^2^ Sackler Faculty of Medicine, Tel Aviv University, Tel Aviv, Israel; ^3^ Rabin Medical Center, Department of Nephrology and Hypertension, Petah-Tikva, Israel; ^4^ Department of Transplantation, Rabin Medical Center, Petah-Tikva, Israel; ^5^ Clinical Microbiology Laboratory, Rabin Medical Center, Beilinson Hospital, Petah-Tikva, Israel; ^6^ Department of Biochemistry and Molecular Genetics, Israel Institute for Biological Research, Ness-Ziona, Israel

**Keywords:** kidney transplant recipients, COVID-19 vaccine, immunosuppression reduction, antibody response, cellular response

## Abstract

Immune response to two SARS-CoV-2 mRNA vaccine doses among kidney transplant recipients (KTRs) is limited. We aimed to evaluate humoral and cellular response to a third BNT162b2 dose. In this prospective study, 190 KTRs were evaluated before and ∼3 weeks after the third vaccine dose. The primary outcomes were anti-spike antibody level >4160 AU/ml (neutralization-associated cutoff) and any seropositivity. Univariate and multivariate analyses were conducted to identify variables associated with antibody response. T-cell response was evaluated in a subset of participants. Results were compared to a control group of 56 healthcare workers. Among KTRs, we found a seropositivity rate of 70% (133/190) after the third dose (37%, 70/190, after the second vaccine dose); and 27% (52/190) achieved levels above 4160 AU/ml after the third dose, compared to 93% of controls. Variables associated with antibody response included higher antibody levels after the second dose (odds ratio [OR] 30.8 per log AU/ml, 95% confidence interval [CI]11–86.4, *p* < 0.001); and discontinuation of antimetabolite prior to vaccination (OR 9.1,95% CI 1.8–46.5, *p* = 0.008). T-cell response was demonstrated in 13% (7/53). In conclusion, third dose BNT162b2 improved immune response among KTRs, however 30% still remained seronegative. Pre-vaccination temporary immunosuppression reduction improved antibody response.

## Graphical Abstract

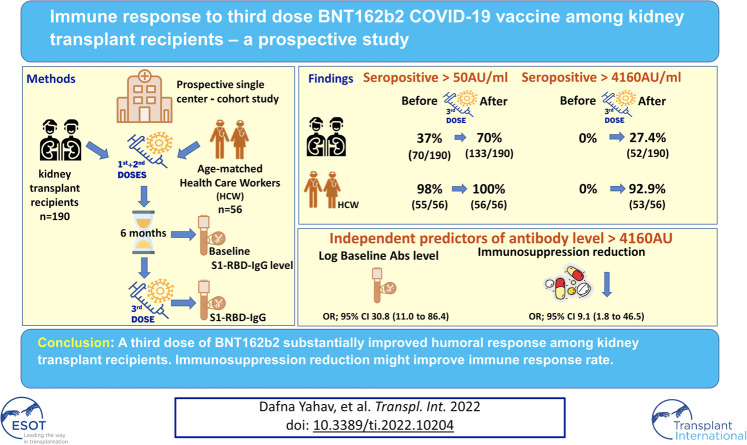

## Introduction

Kidney transplant recipients (KTRs) are at increased risk for severe disease and death from COVID-19, and hence are prioritized for vaccination (1). Several studies evaluating the immune response following a two-dose mRNA vaccine schedule among KTRs demonstrated diminished humoral and cellular response (2–4). Seroconversion rates among KTRs receiving two doses of mRNA vaccine in these studies ranged from 36–54% compared to 100% in healthy controls (2–4). Similarly, T-cell response rates of 30–54% were demonstrated among KTRs, compared to over 95% among healthy controls (4,5). In addition, clinical cases of severe COVID-19, including fatal cases, were reported among fully (two-dose) vaccinated KTRs (6,7). The third mRNA vaccine dose has been recommended for severely immunocompromised patients since April 2021 in France, as well as by the European Medicines Agency (EMA) since October 2021 (1,8). Several previous studies evaluated the effectiveness and safety of a third mRNA vaccine dose among solid organ transplant (SOT) recipients (9–14), with three studies including solely KTRs (9,13,14). Humoral response among SOT recipients was demonstrated in 32–55% of those seronegative after two vaccine doses, without serious adverse events. Cellular response and predictors of negative immune response were partially evaluated (9–14). Immune response to two-dose mRNA vaccines varied between SOT types in previous studies, with KTRs being more responsive than lung transplant recipients, but less responsive than heart and liver transplant recipients (15–17).

In the current study, we aimed to evaluate humoral and cellular response specifically among KTRs ∼3 weeks after a third dose of BNT162b2 vaccine dose in Israel. We also aimed to identify variables associated with positive antibody response.

### Patients and Methods

This is a prospective comparative study conducted in continuation with our previous study, evaluating the effectiveness and safety of a two-dose schedule of BNT162b2 vaccine among KTRs (2). Participants (N = 190) in the current study were consenting KTRs, who participated in the previous study, received a third BNT162b2 vaccine dose (according to the Israeli Ministry of Health recommendation for the entire population, at least 5 months after the second dose), and had antibody levels collected before and after the third dose. These were compared with 56 healthy controls. Vaccines were administered between July 12, 2021 and August 29, 2021 and patients were followed for up to 9 weeks. Participants were scheduled for a study visit ∼3 weeks after the third vaccine dose to collect blood for anti-spike antibody levels and cellular response (See below). Follow up for acute kidney rejection episodes was performed by collecting creatinine levels at the time of antibody levels collection, and requesting that participants report any unusual symptoms. The study was approved by the local ethics committee of the Rabin Medical Center. We collected demographics and data concerning the immunosuppressive medication regimen. Blood samples for anti-spike SARS-CoV-2 antibodies were tested using the SARS-CoV-2 IgG II Quant (Abbott^©^) assay. A test was considered positive when IgG was ≥50 AU/ml (18). Calcineurin inhibitor (CNI) blood levels (tacrolimus or cyclosporine) and creatinine values were also obtained on study visit. Renal function was calculated using the chronic kidney disease epidemiology collaboration (CKD-EPI) equation. T-cell response was measured for 55 randomly selected participants using the SARS-CoV-2 interferon-gamma (IFNg) release assay (EUROIMMUN, Lübeck, Germany) with strict adherence to the manufacturer’s instructions. In this quantitative assay, whole blood was stimulated for 24 h with spike antigen and a control with no antigen. Secreted IFNg in response to stimulation was measured by ELISA (DuoSet, R&D Systems, Minneapolis, MN) and results were presented as the difference between IFNg levels in response to spike versus background response to no antigen control. The results were measured in pg/ml, and a test was considered positive when the difference was >10 pg/ml (weak positive 10–50, positive >50 pg/ml) (19,20).

The primary outcomes were 1) proportion of antibody response above 4160 AU/ml, a threshold that was previously shown to correspond with a 95% probability of viral neutralization (21,22); and 2) any seropositivity (>50 AU/ml) among previously seronegative participants. Secondary outcomes included log transformed antibody levels as a continuous variable, T-cell response, and acute rejection.

### Statistical Analysis

Categorical variables were presented as numbers (percentages), and continuous variables as median (interquartile range, IQR) or mean (SD), according to their distribution. The former was compared using the Chi-square test or Fisher’s exact test and the latter using *t*-test or Mann Whitney test, as appropriate.

Univariate and multivariate logistic regression models were used for the evaluation of variables associated with response (of both >4160 AU/ml, and >50 AU/ml). All variables were considered for inclusion into the multivariate analysis after testing for collinearity using a forward stepwise regression model with a *p* value below 0.05 used for inclusion. Linear regression analyses were performed to explore factors associated with higher log transformed antibody titer among KTRs.

Results were compared with a control group of 56 healthcare workers aged 60–75 years that were immunized with a third BNT162b2 dose during the same period. A General linear model (GLM) was used for comparison of log transformed Ab level between the KTR and control groups with age, gender, creatinine value, body mass index (BMI), and diabetes as covariates using a fixed effect model. Estimated marginal mean (EMM) adjusted for the above variables was calculated to evaluate the adjusted difference of log Ab level with 95% confidence interval. Analyses were performed using IBM SPSS statistics, version 27.

## Results

Of the 308 KTRs in the original cohort (2), 190 (61%) had a baseline anti-spike antibody test collected before the third vaccine dose, and were included in the current study. (See flow chart of patients’ selection in Supplementary Figure S1). Mean age was 59 years (SD 12), and 32% were females (61/190). Median time from third vaccination to antibody test collection was 29 days (IQR 20–33).

### Antibody Response Among KTRs Group

**FIGURE 1:**
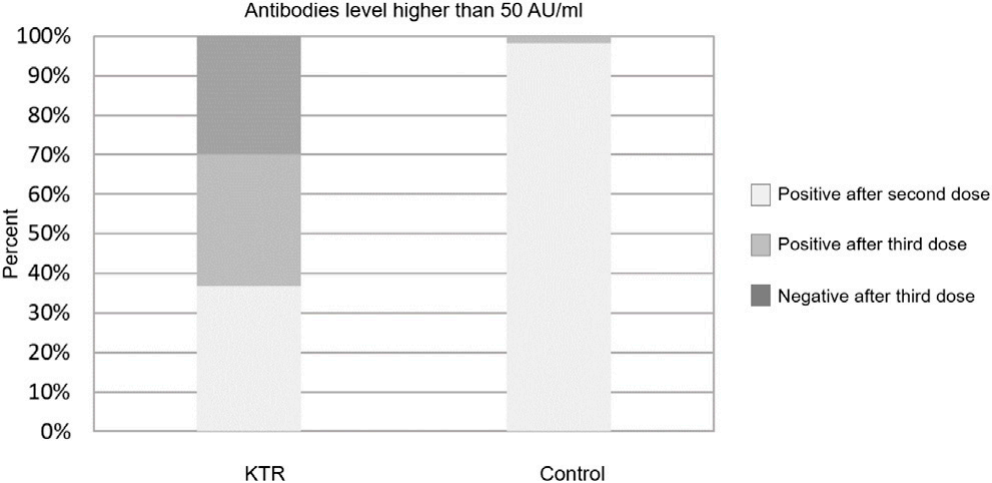
Antibody response rates following second and third dose among kidney transplant recipients and controls–cut-off at 50 AU/ml.

**FIGURE 2:**
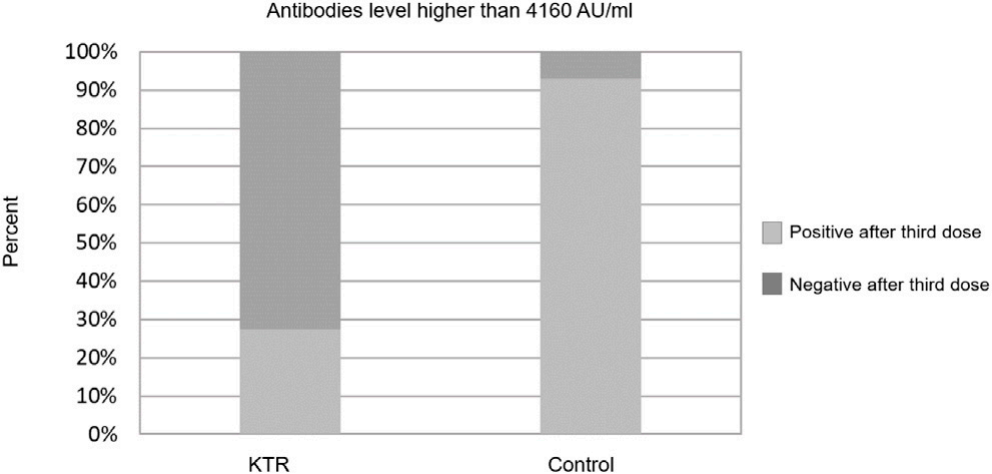
Antibody response rates following second and third dose among kidney transplant recipients and controls–cut-off at 4160 AU/ml.

Overall, 133 (70.0%) KTRs had a positive antibody response (>50 AU/ml) after the third vaccine dose, compared with 70 (36.8%) after the second dose (*p* < 0.001). Sixty three of 120 KTRs (52.5%) were seronegative after second dose but turned seropositive after the third dose (Figure 1). Using a cutoff of 4160 AU/ml, 52 (27.4%) KTRs achieved this antibody level after third dose compared with 52 (92.9%) of the control group (*p* < 0.001). None of the study participants (KTRs or controls) achieved antibody levels >4160 AU/ml after the second vaccine dose (Figure 2).

Characteristics of the study population are presented in Table 1, stratified by antibody response >4160 AU/ml. Twenty-seven KTRs (14.2%) had their immunosuppression reduced permanently or temporarily prior to the third vaccine dose. Among 70% (19/27) of them, antimetabolites were discontinued, usually temporarily; for the other eight KTRs, dose was reduced (reasons for discontinuation and regimens, see Supplementary Table S1).

**TABLE 1:** Characteristics of 190 KTRs, stratified by antibody response >4160 AU/ml.

Variable	All (N = 190)	Response (N = 52, 27%)^a^	No Response (N = 138, 72%)^a^	*p*-value
Age (years) (mean, SD)	59.03 (12.35%)	54.58 (11.86)	60.71 (12.16)	0.002
Female gender (No., percentage)	61 (32.11%)	21 (40.38%)	40 (28.99%)	0.133
Time from transplantation (years) (mean, SD)	7.48 (7.98)	6.62 (6.41)	7.80 (8.49)	0.363
Living donor (No., percentage)	147 (77.37%)	47 (90.38%)	100 (72.46%)	0.008
eGFR (per ml/min/1.73m^2^) (mean, SD)	61.13 (21.48)	70.01 (20.93)	57.78 (20.78)	0.001
Diabetes mellitus (No., percentage)	37 (19.47%)	5 (9.62%)	32 (23.19%)	0.035
Baseline log antibody level (mean, SD)	1.31326 (0.905)	2.34099 (0.515)	0.925999 (0.692)	<0.001
Time from second vaccine dose (days) (mean, SD)	163.38 (1841.01%)	160.96 (2,354.15%)	164.3178 (1,600.00%)	0.275
Immunosuppression reduction (yes) (No., percentage)	27 (14.21%)	9 (17.31%)	18 (13.04%)	0.425
BMI (per kg/m^2^) (mean, SD)	27.22 (4.43)	27.30 (4.12)	27.19 (4.56)	0.877
High antimetabolite dose^b^ (No., percentage)	120 (63.16%)	32 (61.54%)	88 (63.77%)	0.776
High tacrolimus level^c^ (No., percentage)	110 (57.89%)	25 (48.08%)	85 (61.59%)	0.092
mTOR inhibitor (No., percentage)	17 (8.95%)	5 (9.62%)	12 (8.70%)	0.843
Treatment with ATG (No., percentage)	8 (4.21%)	1 (1.92%)	7 (5.07%)	0.335
Cyclosporine use (No., percentage)	30 (15.79%)	10 (19.23%)	20 (14.49%)	0.425

eGFR, estimated glomerular filtration; mTOR, mammalian target of rapamycin; ATG, anti thymocyte globulin.

a Response for this analysis was considered if antibody level increased beyond 4160 AU/ml.

b High antimetabolite dose ≥720 mg per day.

c High tacrolimus level >7 mg/ml.

### Variables Associated With Antibody Response

Univariate analysis for variables associated with antibody response over 4160 AU/ml demonstrated that lower antibody level after the second vaccine dose, older age, lower estimated glomerular filtration rate (eGFR), presence of diabetes mellitus, and transplant from none-living donor were associated with no response (Table 1). Multivariate analysis, introducing immunosuppression reduction into the model, only demonstrated antibody levels after the second vaccine dose and immunosuppression reduction was significantly associated with antibody response (odds ratio [OR] 30.78, 95% confidence interval [CI] 10.97–86.36, *p* < 0.001; and OR 9.06, 95% CI 1.76–46.48, *p* = 0.008, respectively) (Table 2). Performing the same analysis to predict any antibody response (>50 AU/ml) for the 120 nonresponding patients, only baseline antibody level and treatment with cyclosporine (instead of tacrolimus) were demonstrated as significant. (See Supplementary Table S2).

**TABLE 2:** Univariate and multivariate analyses for variables associated with antibody response >4160 AU/ml among 190 KTRs

Variable	Univariate			Multivariate		
	OR	95% CI for OR	p	OR	95% CI for OR	p
Age (per year)	0.961	0.936–0.987	0.003	—	—	—
Female gender	1.660	0.854–3.227	0.135	—	—	—
Time from transplantation (years)	0.980	0.939–1.023	0.362	—	—	—
Living donor	3.572	1.321–9.659	0.012	—	—	—
eGFR (per ml/min/1.73m^2^)	1.029	1.012–1.046	0.001	—	—	—
Diabetes mellitus	0.352	0.129–0.961	0.042	—	—	—
Baseline log antibody level	22.976	9.018–58.540	<0.001	30.78	10.97–86.36	<0.001
Time from second vaccine dose (days)	0.990	0.972–1.008	0.274	—	—	—
Immunosuppression reduction	1.405	0.608–3.244	0.426	9.06	1.76–46.48	0.008
BMI (per kg/m^2^)	1.006	0.936–1.081	0.876	—	—	—
High antimetabolite dose^a^	0.909	0.471–1.755	0.776	—	—	—
High tacrolimus level^b^	0.577	0.303–1.098	0.094	—	—	—
mTOR inhibitor	1.117	0.373–3.341	0.843	—	—	—
Treatment with ATG	0.367	0.044–3.057	0.354	—	—	—
Cyclosporine use	0.542	0.149–1.970	0.352	—	—	—

OR, odds ratio; eGFR, estimated glomerular filtration rate; BMI, body mass index; mTOR, mammalian target of rapamycin; ATG, anti thymocyte globulin.

a High antimetabolite dose ≥720 mg per day.

b High tacrolimus level >7 mg/ml.

### Antibody Response in KTRs Versus Controls

**FIGURE 3:**
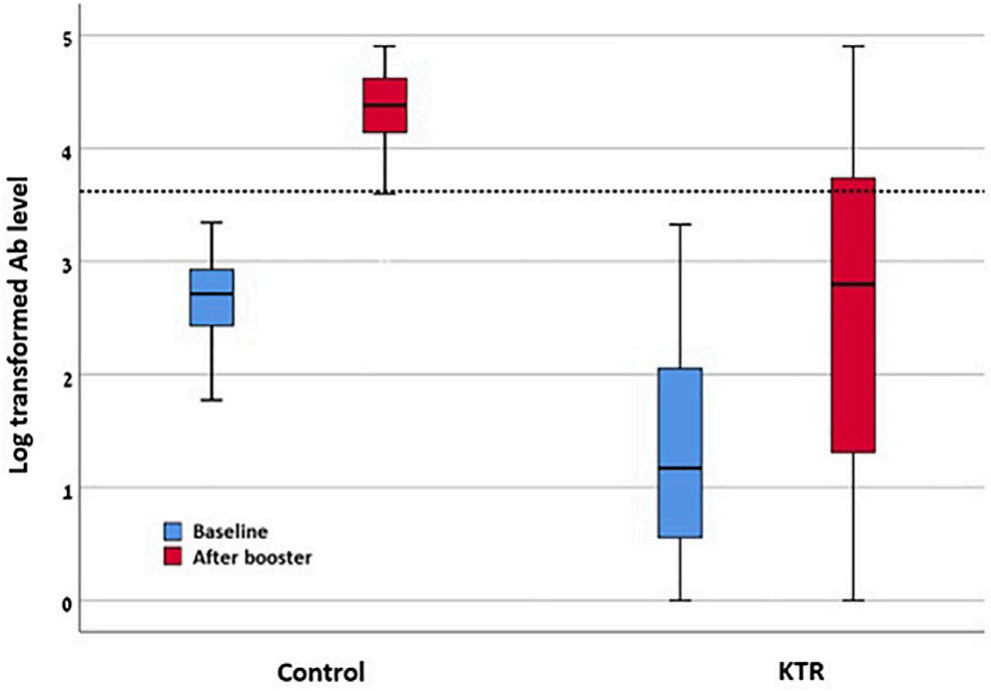
Log transformed antibody levels before and after third vaccine dose among KTR and control. KTR, kidney transplant recipient; “Baseline”—levels as tested after second vaccine dose. “After booster”—after third vaccine dose.

Comparison of the KTR cohort’s baseline characteristics and outcomes versus the healthcare workers control group is detailed in Table 3. Among the control group, 100% were seropositive (>50 AU/ml) after the third dose, while 98% were positive after the second dose. In this group, none of the participants had antibody level >4160 AU/ml prior to the third dose, while this level was achieved in 93% (52/56) after the third dose. Regarding antibody levels achieved, among 190 KTRs, median anti-spike antibody level was significantly increased from 13.8 (IQR 2.6–111.55) AU/ml before, to 514.35 (IQR 19.35–5,474.4) AU/ml after the third dose (*p* < 0.001). Similarly, log transformed antibody level was increased from 1.3 ± 0.9 AU/ml to 2.51 ± 1.37 AU/ml (*p* < 0.001). In comparison, among 56 control group participants, antibody level was increased from 514 (IQR 259.68–857.8) AU/ml before, to 23,800.15 (IQR 259.68–857.8) AU/ml after the third dose. Log antibody level increased from 2.65 ± 0.4 to 4.31 ± 0.42. After adjustment for age, gender, BMI, diabetes mellitus status and creatinine level, the adjusted mean difference of the log transformed antibody level between the control and KTR groups was 1.98 (95% CI 1.57–2.39) AU/ml, reflecting significantly increased antibody levels among the control group. Antibody levels before and after the third vaccine dose are presented in Figure 3.

**TABLE 3:** comparison of the 190 KTRs and 56 controls included in the study.

Variable Name	All		KTR (190)		Control (56)		p
Age (years) (Mean, SD)	61.36	11.940	59.03	12.355	69.27	5.303	<0.001
Female gender (No., percentage)	88	35.77%	61	32.11%	27	48.21%	0.027
Diabetes mellitus (No., percentage)	44	17.89%	37	19.47%	7	12.50%	0.231
BMI (kg/m^2^) (mean, SD)	27.0828	4.229	27.22	4.431	26.60	3.400	0.35
Serum creatinine (mg/dl) (mean, SD)	1.25	0.733	1.36	0.790	0.86	0.207	<0.001
Time to booster dose^a^ (mean, SD)	172.17	23.222	163.38	18.410	201.85	8.468	<0.001
Bassline antibody level (AU/ml) (median, IQR)	52.75	3.68–343	13.80	2.6–111.55	514.35	259.68–857.8	<0.001
Antibody levels after third dose (AU/ml) (median, IQR)	1881.45	59.48–13,299.2	622.40	19.35–5,474.4	23,800.15	13,343–41,511.75	<0.001
Baseline log antibody level (AU/ml) (mean, SD)	1.62	0.99	1.31	0.90	2.65	0.40	<0.001
Log antibody level after third dose (mean, SD)	2.92	1.432	2.51	1.365	4.31	0.417	<0.001
Adjusted log antibody level after third dose^b^ (median, IQR)	—	—	2.32	3.7–4.63	4.17	2.07–2.56	<0.001
Antibody level above 50 AU/ml (No., percentage)	189	76.8%	133	70.0%	56	100.0%	<0.001
Antibody level above 4160 AU/ml (No., percentage)	104	42.3%	52	27.4%	52	92.9%	<0.001

IQR, interquartile range.

a Time between the second and third vaccine dose in days.

b Estimated marginal mean with 95% CI, adjusted for age, gender; BMI, serum creatinine and diabetes mellitus.

### Variables Associated With Higher Titer Antibody Response

When the log transformed antibody level was evaluated as it continued to be variable, the factors that were significantly associated with higher log antibody level were baseline antibody levels before the third vaccine dose, immunosuppression reduction, non-diabetic status, treatment with cyclosporine, and treatment with TOR inhibitors (instead of antimetabolites) (See Supplementary Table S3).

### T-Cell Response

T-cell response, tested in 55 randomly selected KTRs, of whom two were excluded from the analysis because of very high negative control response; of the remaining 53 participants, seven (13.2%) patients had a positive response. This randomly selected subgroup of patients did not differ in baseline characteristics compared to the study population. (Supplementary Table S4 for comparison). Forty patients (75.5%) of the 53 were seropositive after the third vaccine dose.

During the follow up of median 61 days (IQR 56–63), none of the KTRs developed acute graft rejection.

## Discussion

In this study including 190 KTRs, we found 70% seropositivity rates in response to a three dose BNT162b2 regimen, increasing from 37% after two doses. Twenty seven percent (52/190) achieved antibody response over 4160 AU/ml, associated with neutralization. T-cell response rate among KTRs was low, with 7/53 (13%) presenting adequate anti spike T-cell response. Variables associated with antibody response among KTRs included antibody levels after the second vaccine dose and immunosuppression reduction. The antibody response rate documented in our study is in accordance with recent studies reporting improved humoral response 28 days following a third mRNA dose in SOT recipients (9–14). Three of them including solely KTRs: Massa et al. reported antibody response in 32% of 61 seronegative KTRs, improving seropositivity rates from 44% to 62% after three BNT162b2 doses (13). Benotmane et al. reported 49% response among 159 previously seronegative KTRs after three doses of the mRNA-1273 vaccine (9). The relatively increased response rates in the latter study may represent a higher humoral immunogenicity with mRNA-1273 vaccine compared to BNT162b2, previously demonstrated in healthy people (23). Schrezenmeier et al. reported a 36% response rate among 25 previously seronegative KTRs, following either the third homologous BNT162b2 dose or heterologous ChAdOx1 (14). In SOT recipients in general, Kamar et al. reported a 44% response rate among 59 seronegative SOTs, most of them KTRs, with seropositivity rates increasing from 40% to 66% of 101 SOT recipients vaccinated with BNT162b2 (10). Finally, Hall et al. randomized 120 SOT recipients (29 KTRs, 25 kidney-pancreas), 10% seropositive at baseline, to either mRNA-1273 or placebo. Fifty five percent of 60 patients in the vaccine arm were seropositive after the third dose, compared with 18% in the placebo group (11). Magnitude of response was also demonstrated to increase after third dose, similar to our study (9,24).

Surprisingly, using the cutoff of 4160 AU/ml, the control group in our study was seronegative prior to the third dose. The mean age of participants in the control group was 69 years, and they were on average 200 days after the second vaccine. Waning of vaccine response has been demonstrated, mainly in older adults, which may explain the relatively low antibody titer (25). In addition, the cutoff used as surrogate for neutralization in our study was taken from previous studies. Additional studies may be needed to validate this cutoff as a surrogate for neutralization.

We found in our cohort 13% (7/53) of patients with adequate T-cell response, evaluated by SARS-CoV-2 spike-specific IFNg secretion. These rates are lower than the 30–60% cellular response rates reported after second vaccine dose among KTRs, measured by IFNg secreting cells frequency, which is of higher sensitivity than IFNg secretion assay (4). A significant increase in IFNg secreting cells was demonstrated in studies evaluating SOT recipients before and after the third vaccine dose (11,13,14). Percentage of responders was not reported in these studies, and assays differed, limiting our ability to compare them to our results. In a small study in cancer patients, no improvement in T-cell response was observed after the BNT162b2 third dose (26). Schrezenmeier et al. reported that KTRs with a humoral response to the third vaccine dose had significantly higher portions of antigen-reactive T cells than those without a humoral response (14).

Various predictors of antibody response to the third mRNA dose were reported from previous studies in SOT recipients. Among KTRs, use of antimetabolite, low lymphocyte count, and previous negative antibody response to the second vaccine dose predicted negative response (9). In SOT recipients in general, older age, higher degree of immunosuppression, and a lower estimated glomerular filtration rate were associated with no antibody response (10). Previous studies assessing immune response to second mRNA vaccine dose in KTRs demonstrated mycophenolate including regimen as strongly associated with low seroconversion rates (2,3). In the study by Schrezenmeier et al. only three individuals achieved high positive antibodies, one of them was the only person in the study without mycophenolate mofetil at the time of vaccination (14).

These findings may explain our results, showing immunosuppression reduction prior to vaccination to be associated with positive antibody response.

Following the third vaccine dose and although immunosuppression was discontinued for 14.2% of the cohort, no acute rejection episodes were found in our cohort. Previous studies demonstrated no serious adverse events and no acute rejection episodes among SOT recipients who received a third mRNA vaccine dose (9–11,24). In addition, previous studies have demonstrated no graft or patient survival impairment following temporary discontinuation of mycophenolate mofetil in KTRs treated with triple immunosuppression, as in our study (27,28).

Our study has several limitations. The sample size was limited with a small number of events included in the multivariable analysis. Cellular response was tested only after the third vaccine dose, with no previous testing after second dose for comparison. It was also assessed only in a limited subset of patients due to technical limitations. In addition, neutralizing antibodies were not directly assessed. Nevertheless, we used a cut-off of 4160 AU/ml of anti-spike antibodies as a surrogate, as previously described (21,22). Hall et al. demonstrated significant median percent virus neutralization (71%) with a third dose versus placebo (13%); and Massa et al. also demonstrated a significant increase in serum neutralizing activity between the second and third doses (11,13). The significance of higher antibody response as reflecting protection against infection and severe disease is still debated, though data are accumulating to support an association. A recent study from Israel demonstrated among healthcare workers both higher antibody levels and recused risk for infection after third versus second BNT162b2 dose. Greater incidence of infection was demonstrated among those with lower antibody levels (29). Immunosuppression reduction was not planned for the study and was initiated by either patients or treating physicians, due to reasons related or unrelated to the vaccine. This strategy should be tested in clinical trials, designated for this question (30).

Though our results and others present improved humoral immunity following three vaccine doses, still, at least 30% of KTRs remain seronegative, potentially susceptible to SARS-CoV-2 infection. These results support the administration of a third vaccine dose to KTRs; however, additional strategies should be discussed. These may include immunosuppression reduction prior to vaccination (30). Considering that anti-metabolites are consistently reported as associated with seronegative response, transient discontinuation of anti-metabolites prior to and shortly after the vaccine dose may be considered, monitoring meticulously for acute rejection (14). An alternative approach could be heterologous vaccination, i.e., using a non-mRNA vaccine for the third dose. Such a “mix and match” approach was recently supported by the EMA for the general population, stating that there are currently no data to support heterologous boosting among immunocompromised individuals (31). Two studies in KTRs used heterologous third dose boosting, with approximately a third to half of the recipients developing humoral response (12,14). This approach should be further tested in this population. An additional approach could be a fourth mRNA vaccine dose. Three studies evaluating a fourth vaccine dose among KTRs have been published so far, demonstrating improved immunogenicity, though with lower response rates among those still seronegative after the third dose (32–34). Otherwise, consideration of post-exposure or even pre-exposure prophylaxis could be an alternative to vaccination, using monoclonal antibodies or antiviral drugs (35–38). In addition, safety results should be verified in larger, longer term follow-up studies, including follow-up on rejection risk (39).

In summary, third dose BNT162b2 improves immune response over two doses in KTRs; however a significant percentage of these patients still remain seronegative and at risk for SARS-CoV-2 infection. Temporary withdrawal of the antimetabolite should be considered before the administration of the vaccine dose, taking into account individual risk for rejection. This strategy and others for improved immunization should be tested in clinical studies.

## Data Availability

The raw data supporting the conclusion of this article will be made available upon reasonable request to the authors.

## Abbreviations

CNI, Calcineurin inhibitor; IFNg, interferon-gamma; KTRs, Kidney transplant recipients; SOT, Solid organ transplant.
