# Supplementary material for: Immune Response to Third Dose BNT162b2 COVID-19 Vaccine Among Kidney Transplant Recipients—A Prospective Study
Source: Transpl Int. 2022 Apr 21;35:10204. doi: 10.3389/ti.2022.10204 (PMC9068869; doi:10.3389/ti.2022.10204)
Supplement: Supplementary file 1 [file DataSheet1.docx]

**Supplemental Table 1: Reasons and schedule for anti-metabolite changes**

| **Serial number** | **Age** | **Gender^1^** | **Dose^2^** | **High CNI level^3^** | **Baseline Ab level (AU/ml)^4^** | **Discontinuation or reduction (D or R)** | **Reason** | **Timing** |
| --- | --- | --- | --- | --- | --- | --- | --- | --- |
| 1 | 55 | m | 2 | 1 | 494 | D | BK viremia | More than 30 d before booster |
| 2 | 53 | f | 3 | 1 | 26 | R | Adverse event | More than 30 d before booster |
| 3 | 59 | m | 3 | 1 | 0 | D | BK viremia | More than 30 d before booster |
| 4 | 41 | f | 2 | 1 | 88 | D | Vaccination | During the week before vaccine |
| 5 | 74 | f | 2 | 1 | 2 | D | Vaccination | During the week before vaccine |
| 6 | 68 | m | 3 | 1 | 95 | D | Vaccination | During the week before vaccine |
| 7 | 48 | f | 2 | 0 | 822 | D | Vaccination | During the week before vaccine |
| 8 | 69 | m | 2 | 0 | 2 | D | Vaccination | During the week before vaccine |
| 9 | 43 | m | 2 | 1 | 23 | D | Adverse event | More than 30 d before booster |
| 10 | 52 | m | 3 | 1 | 10 | D | Vaccination | During the week before vaccine |
| 11 | 62 | m | 1 | 1 | 0 | D | NS | NS |
| 12 | 45 | m | 1 | 1 | 0.5 | D | Vaccination | During the week before vaccine |
| 13 | 40 | m | 3 | 1 | 0.5 | D | Vaccination | During the week before vaccine until 5d after |
| 14 | 55 | f | 3 | 1 | 2 | D | Vaccination | During the week before vaccine |
| 15 | 64 | m | 3 | 1 | 80 | D | Vaccination | During the week before vaccine |
| 16 | 58 | m | 2 | 1 | 0 | D | BK viremia | More than 30 d before booster |
| 17 | 55 | f | 3 | 1 | 5 | R | Adverse event | More than 30 d before booster |
| 18 | 74 | m | 2 | 0 | 16 | R | NS | NS |
| 19 | 61 | f | 2 | 1 | 0 | D | Vaccination | During the week before vaccine |
| 20 | 64 | f | 3 | 0 | 0 | D | Vaccination | During the week before vaccine |
| 21 | 67 | m | 2 | 1 | 56 | D | Vaccination | During the week before vaccine |
| 22 | 62 | m | 2 | 1 | 3 | D | Vaccination | During the week before vaccine |
| 23 | 58 | m | 2 | 0 | 2 | D | Vaccination | During the week before vaccine |
| 24 | 52 | 2 | 2 | 0 | 420 | D | Vaccination | During the week before vaccine |
| 25 | 53 | m | 2 | 1 | 121 | D | Vaccination | During the week before vaccine |
| 26 | 66 | m | 3 | 1 | 1 | D | Vaccination | During the week before vaccine |
| 27 | 47 | m | 2 | 1 | 1 | D | Vaccination | During the week before vaccine |

NS – not specified

**^1^** Gender: m – male; f - female

**^2^** Dose – number of mycophenolate mophetil tablets (360 mg) 1at baseline

**^3^** High dose – tacrolimus level > 7 ng/mL

**^4^** Positive test considered > 50 AU/ml (i.e., 19 patients seronegative)

**Supplemental Table 2: Univariate and multivariate analyses for variables associated with antibody response (> 50 AU/ml) among 120 KTRs with negative antibody level at baseline**

|  | **Univariate** | | | | **Multivariate** | | | |
| --- | --- | --- | --- | --- | --- | --- | --- | --- |
| **Variable** | **OR** | **95% CI for OR** | | **p** | **OR** | **95% CI for OR** | | **p** |
| Age (per year) | 0.997 | 0.967 | 1.029 | 0.857 | - | - | - | - |
| Female gender | 0.891 | 0.410 | 1.937 | 0.771 | - | - | - | - |
| Time from transplantation | 1.025 | 0.982 | 1.069 | 0.256 | - | - | - | - |
| Living donor | 1.098 | 0.484 | 2.488 | 0.824 | - | - | - | - |
| eGFR (per ml/min/1.73m^2^) | 1.013 | 0.995 | 1.031 | 0.163 | - | - | - | - |
| Diabetes mellitus | 0.425 | 0.176 | 1.027 | 0.057 | - | - | - | - |
| Baseline log antibody level | 10.229 | 3.903 | 26.806 | 0.000 | 14.726 | 5.017 | 43.229 | 0.000 |
| Time from second vaccine dose | 1.000 | 0.975 | 1.024 | 0.968 | - | - | - | - |
| Immunosuppression reduction | 0.967 | 0.362 | 2.581 | 0.947 | - | - | - | - |
| BMI (per kg/m^2^) | 0.990 | 0.914 | 1.071 | 0.794 | - | - | - | - |
| High antimetabolite dose* | 0.624 | 0.290 | 1.344 | 0.228 | - | - | - | - |
| High tacrolimus level** | 1.145 | 0.542 | 2.419 | 0.722 | - | - | - | - |
| mTOR inhibitor | 4.418 | 0.912 | 21.397 | 0.065 | - | - | - | - |
| Treatment with ATG | 0.869 | 0.168 | 4.488 | 0.867 | - | - | - | - |
| Cyclosporine use | 2.407 | 0.710 | 8.158 | 0.158 | 6.563 | 1.516 | 28.410 | 0.012 |

OR – odds ratio; eGFR – estimated glomerular filtration rate; ATG – anti thymocyte globulin

* High antimetabolite dose ≥ 720 per day

** High tacrolimus level > 7 mg/ml

**Supplemental Table 3: Univariate and multivariate analyses for variables associated with higher log antibody level**

|  | **Univariate** | | | | **Multivariate** | | | |
| --- | --- | --- | --- | --- | --- | --- | --- | --- |
| **Variable** | **B** | **95% CI for B** | | **p** | **B** | **95% CI for B** | | **p** |
| Age (per year) | -0.025 | -0.040 | -0.009 | 0.002 | - | - | - | - |
| Female gender | -0.033 | -0.453 | 0.386 | 0.876 | - | - | - | - |
| Time from transplantation | -0.001 | -0.026 | 0.023 | 0.916 | - | - | - | - |
| Living donor | 0.499 | 0.036 | 0.961 | 0.035 | - | - | - | - |
| eGFR (per ml/min/1.73m^2^) | 0.017 | 0.008 | 0.026 | 0.000 | - | - | - | - |
| Diabetes mellitus | -0.713 | -1.197 | -0.229 | 0.004 | -0.320 | -0.602 | -0.037 | 0.027 |
| Baseline log antibody level | 1.228 | 1.102 | 1.354 | 0.000 | 1.240 | 1.114 | 1.367 | 0.000 |
| Time from second vaccine dose | -0.002 | -0.013 | 0.009 | 0.737 | - | - | - | - |
| Immunosuppression reduction | -0.180 | -0.717 | 0.356 | 0.509 | 0.326 | 0.017 | 0.635 | 0.039 |
| BMI (per kg/m^2^) | 0.008 | -0.037 | 0.052 | 0.733 | - | - | - | - |
| High antimetabolites dose* | -0.173 | -0.336 | -0.010 | 0.038 | - | - | - | - |
| High tacrolimus level** | -0.383 | -0.775 | 0.010 | 0.056 | - | - | - | - |
| mTOR inhibitor | 0.565 | -0.117 | 1.246 | 0.104 | 0.458 | 0.066 | 0.851 | 0.022 |
| Treatment with ATG | -0.280 | -1.254 | 0.695 | 0.572 | - | - | - | - |
| Cyclosporine | -0.251 | -0.937 | 0.434 | 0.470 | 0.461 | 0.067 | 0.855 | 0.022 |

eGFR – estimated glomerular filtration rate; ATG – anti thymocyte globulin

* High antimetabolite dose ≥ 720 per day

** High tacrolimus level > 7 mg/ml

**Supplemental Table 4: Baseline characteristics of 53 individuals tested for T-cell response compared with the other study population**

| **Variable name** | **All** | | **Evaluated (53p)** | | **Not evaluated (137p)** | | **p** |
| --- | --- | --- | --- | --- | --- | --- | --- |
| Age (year) (mean, SD) | 59.03 | 12.35 | 56.49 | 13.49 | 60.02 | 11.79 | 0.077 |
| Female gender (No., percentage) | 61 | 32.11% | 17 | 32.08% | 44 | 32.12% | 0.996 |
| Time from transplantation (years) (mean, SD) | 7.48 | 7.98 | 7.73 | 8.33 | 7.38 | 7.86 | 0.786 |
| Living donor (No., percentage) | 147 | 77.37% | 41 | 77.36% | 106 | 77.37% | 0.998 |
| eGFR (per ml/min/1.73m2) (mean, SD) | 61.13 | 21.48 | 61.00 | 20.34 | 61.17 | 21.97 | 0.961 |
| Diabetes mellitus (No., percentage) | 37 | 19.47% | 8 | 15.09% | 29 | 21.17% | 0.343 |
| Time from second vaccine dose (days) (mean, SD) | 163.38 | 18.41 | 164.08 | 18.77 | 163.10 | 18.33 | 0.75 |
| Immunosuppression reduction (No., percentage) | 30 | 15.79% | 5 | 9.43% | 25 | 18.25% | 0.135 |
| BMI (per kg/m2) (mean, SD) | 27.22 | 4.43 | 26.48 | 4.09 | 27.50 | 4.54 | 0.154 |
| High antimetabolites dose (No., percentage) | 120 | 63.16% | 33 | 62.26% | 87 | 63.50% | 0.874 |
| Higher tacrolimus level (No., percentage) | 110 | 57.89% | 33 | 62.26% | 77 | 56.20% | 0.448 |
| mTOR inhibitor (No., percentage) | 17 | 8.95% | 4 | 7.55% | 13 | 9.49% | 0.674 |
| High dose corticosteroids (No., percentage)* | 16 | 8.42% | 5 | 9.43% | 11 | 8.03% | 0.754 |
| Treatment with ATG (No., percentage) | 8 | 4.21% | 3 | 5.66% | 5 | 3.65% | 0.536 |
| Cyclosporine (No., percentage) | 17 | 8.95% | 5 | 9.43% | 12 | 8.76% | 0.884 |

eGFR – estimated glomerular filtration rate; ATG – antithymocytic globulins

* High dose steroids use was defined as use of ≥20 mg prednisone for over 2 weeks at any time after the second vaccine dose
